# Risk stratification by systemic manifestations secondary to hemodynamic disorders of patients with severe tricuspid regurgitation

**DOI:** 10.1186/s12872-024-03805-2

**Published:** 2024-03-12

**Authors:** Xing-Yu Ji, Lei Zhu, Fei Chen, Fang-Lin Lu, Yuan Feng, Mao Chen, Tian-Yuan Xiong

**Affiliations:** 1 Laboratory of Cardiac Structure and Function, Institute of Cardiovascular Diseases, West China Hospital, Sichuan University, 37 Guoxue Alley, Chengdu, 610041 China; 2 Department of Cardiology, The Second Hospital of Tianjin Medical University, Tianjin, China; 3 Department of Cardiovascular Surgery, The First People’s Hospital, Shanghai Jiaotong University, Shanghai, China

**Keywords:** Tricuspid regurgitation, Systemic manifestations, Extra-cardiac manifestations

## Abstract

**Background:**

Tricuspid regurgitation (TR) is a prevalent disease that triggers systemic pathological changes including cardiac, respiratory, hepatic and digestive, hematopoietic, renal and skin issues. The burden of extra-cardiac manifestations has not been well described in TR patients and the clinical impact is unknown.

**Methods:**

Patients with severe or more-than-severe TR during hospitalization, who did not have any previous cardiac procedures, hemodynamically significant congenital heart disease or concomitant severe aortic or mitral valve disease, were retrospectively analyzed. Pre-specified criteria and diagnosis of baseline characteristics were used to evaluate the presence of extra-cardiac manifestations secondary to TR after excluding comorbidities that may also lead to corresponding abnormalities. Extra-cardiac involvements encompass respiratory, hepatic and, digestive, renal, hematopoietic and dermatic system. Staging criteria are defined as no extra-cardiac system involvement in Stage 1, one in Stage 2, at least two extra-cardiac involvements in Stage 3 and any end-stage organ failure in Stage 4. A telephone follow-up was conducted to record the composite endpoint namely all-cause death or cardiac rehospitalization after the index hospitalization.

**Results:**

A total of 258 patients were identified with a median age of 73 (interquartile range [IQR]: 62–83) years and 52.3% were female. Severe TR and more-than-severe TR patients accounted for 92.6% and 7.4% of the cohort. There were 20.5%, 27.5%, 37.6% and 14.3% of patients from Stage 1 to 4 respectively. The follow-up time was at a median of 251 (IQR: 183–324) days. TR Patients in Stage 3&4 were at an increased risk with borderline statistical significance to experience the composite endpoint compared to patients in Stage 1&2 (odds ratio [OR] 1.9, 95% confidence interval [CI] 1.0 to 3.7, *P* = 0.049).

**Conclusions:**

Approximately half of patients with at least severe TR presented with two or more extra-cardiac systemic manifestations, which may incur a 1.9-fold higher risk of all-cause death or cardiac rehospitalization than TR patients with one or less extra-cardiac involvement.

## Highlights

**Key findings**

The presence of ≥2 extra-cardiac manifestations impairs prognosis in TR.

**What is known and what is new?**

In-hospital patients with at least severe TR have variable presentations.

Half of patients with severe and more-than-severe TR presented with two or more extra-cardiac systemic abnormalities.

**What is the implication, and what should change now?**

The comorbid status of patients with severe TR should be systematically assessed to provide a preference for timely intervention.

## Introduction

Tricuspid regurgitation (TR) is a prevalent disease, with a prevalence of 2.7% for moderate or severe TR in individuals aged ≥ 65 years in a primary care population [1]. TR shows a poor prognosis which has been associated with increased mortality independent of pulmonary pressure or right heart failure [2]. Unfortunately, less than 0.5% of cases received surgery [3] and approximately 40% of patients underwent isolated TR surgery during nonelective admission while a significant proportion of patients diagnosed with severe TR were not provided with any definitive treatments [4]. Due to its relatively long disease course, the hemodynamic disorders secondary to TR constantly involve other systems in advanced stage including cardiac, respiratory, hepatic and digestive, hematopoietic, renal and skin issues that express different presentations, inducing repeated hospitalizations and impaired quality of life. Nevertheless, extra-cardiac manifestations have not been well described in TR patients. A substantial number of isolated TR patients exhibit concurrent chronic kidney disease (23%) and liver disease (11%), whose etiology are not specified as secondary to TR or other diseases [4]. As advance in catheter-based valve therapies, less invasive treatment options currently have been offered to undertreated tricuspid valve, making the depiction of the potential candidates and the timing of intervention increasingly critical. It is now recognized that early therapy of TR patients is essential to guarantee benefits from interventions for favorable outcomes [5]. Besides structural or functional indicators within the cardiac system for TR patients, a systemic patient-centered risk stratification may add value to earlier referrals and comprehensive assessments.

We have previously proposed the notion of TR syndrome to stage patients according to their systemic manifestations to provide a reference for timely intervention in TR patients [6] and we would like to elaborate on that staging strategy. In this retrospective analysis, we sought to provide the prevalence of severe or greater TR in the overall patient pool, describe clinical characteristics of these patients, stage patients by different burden of extra-cardiac involvements likely secondary to TR and explore the clinical impact of these systemic manifestations.

## Methods

### Patient population

A retrospective search of in-hospital patients with an echocardiographic diagnosis of moderate or greater TR from January to September 2021 in West China Hospital, Sichuan University was firstly performed. This hospital is a high-volume institution of excellence with 283,000 in-hospital visits in 2021. Additionally, patients with at least severe TR who did not have any previous cardiac procedures or hemodynamically significant congenital heart disease were further selected. Patients with concomitant severe-grade aortic or mitral valve disease were excluded to highlight the impact of TR. Electronic medical records and laboratory or imaging examinations from the corresponding hospitalization were retrieved. The study was approved by our institutional review board with a waiver to obtain patient informed consent.

### Echocardiographic assessment

Echocardiographers grade TR in accordance with the American Society of Echocardiography guidelines [7]. Although a new classification of TR, including massive and torrential beyond severe, has been proposed [8], it has not been used constantly across different echocardiographers in our hospital. However, when TR is more than severe, the echocardiograph report will generally leave comments on the excessive severity. Thus, we analyzed our patients in severe TR and more-than-severe TR subgroups. Duplicate echocardiographic records of the same patient were excluded to keep the earliest examination during the index hospitalization.

### Stage of systemic manifestations

**Fig. 1:**
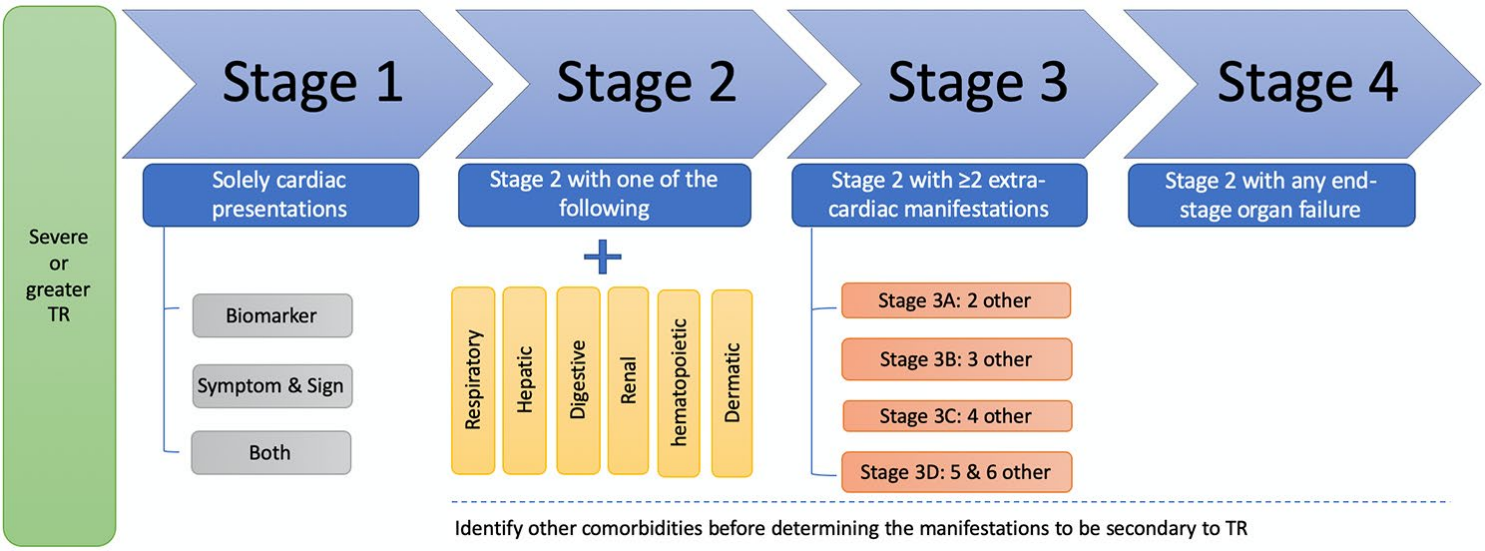
Classification of extra-cardiac involvement in patients with severe TR

The symptomatic progression of TR commences with pulmonary and central venous congestion and culminates in chronic right heart failure, thus, we propose the respiratory, hepatic and digestive, renal, hematopoietic and dermatic system will be prone to be affected by TR. For cardiac systemic involvement, we collected echocardiographic measurements, the value of high-sensitivity troponin T (hs-TnT, > 14 ng/L) and N-terminal-proB-type natriuretic peptide (NT-proBNP, > 334 ng/L), as well as typical symptoms and signs of heart failure in medical records including orthopnoea, paroxysmal nocturnal dyspnea, reduced exercise tolerance, fatigue, tiredness, increased time to recover after exercise, hepatojugular reflux, gallop rhythm, and laterally displaced apical impulse [9]. To determine whether a certain extra-cardiac system was impacted, with consideration of available materials, we used the following criteria to evaluate, (I) respiratory system, pulmonary edema on chest computed tomography (CT); (II) hepatic function, the combination of laboratory abnormalities in values of bilirubin (> 28 µmol/L), alanine aminotransferase (ALT, > 50 IU/L), aspartate transaminase (AST, > 40 IU/L) and albumin (< 40 g/L), in additional to presentations of ascites and hepatomegaly [9] on abdomen CT or echocardiography; (III) digestive system, intestinal wall edema on abdominal CT, or related symptoms and signs including nausea, abdominal fullness [9], or the use of related medications during hospitalization (except treatments for constipation, proton pump inhibitors used for peptic ulcer and antiemetric agents after certain treatments); (IV) renal system, the value of creatinine (> 108 µmol/L in male and > 79 µmol/L in female) and estimated glomerular filtration rate (eGFR, < 56 ml/min/1.73 m^2^); (V) hematopoietic system, congestive splenomegaly on abdominal CT, or pancytopenia (the count of red blood cells < 4.3 × 10^12^/L in male and < 3.8 × 10^12^/L in female, white blood cells < 3.5 × 10^9^/L and platelets < 100 × 10^9^/L, with the value of hemoglobin < 130 g/L in male and < 115 g/L in female); (VI) dermatic system, related symptoms and signs in medical records including pruritus, scratch marks, pigmentation, ulceration. If multiple tests were performed, we chose the worst result. All the included patients were evaluated by the same physician (L. Z.) to maintain consistency. When the evidence was absent during index hospitalization, we marked ‘unable to determine’ to the corresponding system. We proposed a 4-stage scheme to group TR patients (Fig. 1), i.e. (I) Stage 1, solely cardiac presentations; (II) Stage 2, one extra-cardiac involvement; (III) Stage 3, at least two extra-cardiac system involvements, from Stage 3 A to Stage 3D alternatively represents 2, 3, 4, 5 and 6 other systemic manifestations; (IV) Stage 4, any end-stage organ failure.

In addition, the available evidence was classified into 3 levels, i.e. definitive laboratory or imaging examinations (class I), suggestive hints such as the use of medications or signs during physical examinations (class II) and self-reported symptoms (class III).

### Determination of the reason for abnormalities

According to the requirements of medical insurance, the discharge diagnosis was regulated to be complete, thus we used this item to determine whether there were reasons other than TR that could be responsible for extra-cardiac involvements. If other explanations could be identified for a certain abnormality, the patient would not be marked as affected in that system due to TR.

### Follow-up of the cohort

Patients with at least severe TR during hospitalization between January and September 2021 were retrospectively enrolled in this study. A telephone follow-up was conducted during the week of 17th January, 2022. Survival status and events of rehospitalization were inquired but the exact date of events could often not be provided. The composite endpoint was all-cause death or cardiac rehospitalization (including any type of cardiac reasons) after the index hospitalization. Patients who were unable to be contacted by telephone call were excluded from the follow-up analysis.

### Statistical analysis

Categorical variables were presented as frequencies and percentages and continuous variables were presented as the mean ± standard deviation or median with the 25th and 75th percentiles. The Kruskal-Wallis H tests was used for continuous variables, while the Chi square test, Fisher’s exact test and Mann-Whitney U test were used for comparing categorical variables among different stages. A Chi-square test was performed to compare Stage 1&2 vs. Stage 3&4 on the composite primary endpoint (stages were merged for comparison due to a relatively small sample size). All computations relied on commercially available software (SPSS Statistics version 26, IBM Corp, Armonk, New York), and the statistical significance was set at two-tailed 0.05.

## Results

During the targeted 9 months, a total of 138,670 records of echocardiograms were retrieved, among which 1449 records documented moderate or greater TR. A total of 258 patients with at least severe TR were identified. The median age in this cohort was 73 (IQR: 62–83) years and 52.3% were female (Table 1). Severe TR and more-than-severe TR patients accounted for 92.6% and 7.4% of the cohort respectively. Concomitant less than severe aortic or mitral valve disease was alternatively present in 162 (62.8%) and 75 (29.1%) patients. Among the 95 patients whose tricuspid annular plane systolic excursion was measured, the mean value was 14.9 ± 5.2 mm. Tests of hs-TnT and NT-proBNP were available in 237 and 240 patients, with median values of 20.3 (IQR: 13.1–35.3) ng/L and 1677 (IQR: 930–3309) ng/L. Severe or greater pulmonary arterial hypertension was recorded in 33.3% of the 144 patients who had echocardiographic pulmonary artery pressure data. Around a third of patients (33.7%) were admitted to the department of cardiology. The collective representation of other departments, totaling approximately 40% of the patients, encompassing geriatrics (13.2%), cardiovascular surgery (10.5%), nephrology (5.8%), pulmonary medicine (5.4%), and gastroenterology (4.7%).

**Fig. 2:**
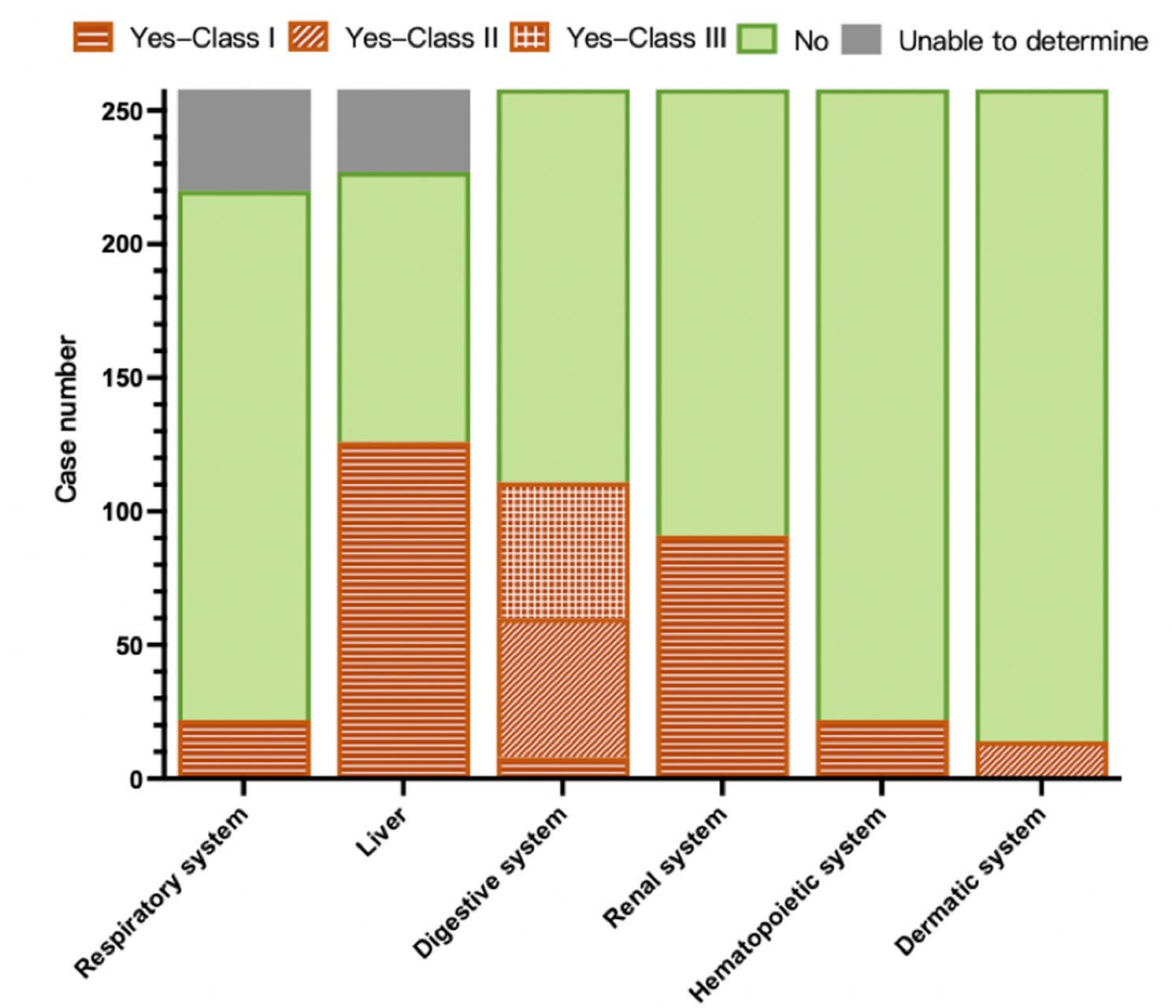
Detailed distribution of abnormalities with level of evidence

The chief complaint at admission was related to the cardiovascular system in 170 (65.9%) patients. There were 22.9% of patients in Stage 1 with no extra-cardiac manifestation. The proportion of patients grouped as having certain extra-cardiac manifestations was illustrated in Fig. 2, with the liver most likely being affected (49.8% of patients). The percentage of patients with no to five extra-cardiac systems affected was 22.9%, 30.6%, 24.8%, 18.6%, 1.9% and 1.1% respectively, and no patient experienced the involvement of all five extra-cardiac systems. A total of 37 (14.3%) patients were diagnosed with organ failure, 45.9% of whom had heart failure. Taken together, there were 20.5%, 27.5%, 37.6% and 14.3% of patients from Stage 1 to 4, respectively.

The follow-up was done in 213 (82.6%) patients at a median of 251 (IQR: 183–324) days after discharge, including 194 patients with severe TR and 19 patients with more-than-severe TR. Only 2 patients reported definitive treatments during follow-up (one transcatheter edge-to-edge repair and one heart transplantation). Altogether 47 patients had the composite primary endpoint, among whom 40 (18.8%) patients were in the group of severe TR while 7 patients were in the group of more-than-severe TR. There was no significant difference in the occurrence of endpoint events between patients with different TR severity (*p* = 0.104), the distribution of endpoints by different stages was shown in Table 1. TR Patients in Stage 3&4 were at higher risk with borderline statistical significance to experience the composite endpoint than patients in Stage 1&2 (OR 1.9, 95% CI 1.0 to 3.7, *P* = 0.049).

## Discussion

This study provides the little evidence existed to understand the comorbid status of the patient population with at least severe TR in a hospital setting, who might be potential candidates for either surgery or transcatheter intervention. The major findings are as follows: (I) Severe or greater TR is prevalent and more than half of the admission was to departments other than cardiology and cardiovascular surgery; (II) Approximately 50% of severe TR patients presented with abnormalities in two or more extra-cardiac systems, indicating a heavy burden of hemodynamic disorders from TR; (III) TR patients in severe or above grades represented no significant difference in the occurrence of composite endpoints; (IV) The staging system we proposed to systematically evaluate extra-cardiac involvements from TR might distinguish patients at higher occurrence of adverse outcomes, with a 1.9-fold risk with borderline statistical significance in Stage 3&4 compared to Stage 1&2.

Tricuspid regurgitation is commonly identified and its incidence increases with age [10]. The prevalence of TR was similar to the aortic stenosis in a community-based analysis [11]. TR has been proved to be associated with increased mortality regardless of pulmonary pressure or right heart failure [2]. Risk stratification of long-term mortality has been done in patients with severe TR that mainly emphasizes on echocardiographic quantification and measurements [12]. The prognosis of TR is not satisfactory, whereas it shows that only 8000 patients undergoing tricuspid valve surgery per year in the United States that has been estimated to own at least 1.6 million severe or greater TR patients [13]. The cause of this unmet need is multifactorial. It is previously believed that functional TR will improve after the correction of valvular disease of the left heart which accounts for the conservative treatment of TR. Although the fate of TR after left-sided valve surgery is conflicting [14, 15], guidelines have recommended concurrent treatment of severe TR together with left-sided valve surgery [16] since the shape and function of the tricuspid valve can not reliably return to baseline and reoperation for isolated TR is associated with significant in-hospital mortality [17]. On the other hand, isolated TR surgery is indicated for patients with severe TR who are either symptomatic or have progressive right ventricular dysfunction, whereas the in-hospital mortality has been reported to reach 8–10% [4, 18] which might increase the further reluctance of offering definitive treatments to TR patients. Earlier surgery for isolated TR has been suggested by the finding that the patients without class I or endocarditis indications had superior survival compared to patients with these indications [19], but how to judge the timepoint remains unclear.

The current profile of TR patients referred to definitive procedures seems too absolute. According to the Nationwide Inpatient Sample database, patients undergoing surgical tricuspid valve replacement from 2011 to 2014 (63.9% in combination with other cardiac procedures) had an incidence of chronic pulmonary disease of 18%, anemia of 26.1% and chronic renal disease of 27.7% [4]. Patients in the TriValve Registry examining the applications of transcatheter tricuspid valve intervention showed similar high-risk profile, with the prevalence of previous admission for right ventricular failure of 69%, an incidence of chronic pulmonary disease of 78%, a mean eGFR of 42.6 ± 18.5 ml/min and a median AST/ALT of 29/20 UI/L. In such a morbid patient population, dedicated risk factors or risk score models were developed to predict the outcome of tricuspid valve intervention while mainly concentrated on anatomical, morphological or functional assessments of the heart [20–22]. In recent years, a research has raised the TRI-SCORE model, namely 6 out of 8 parameters rely on assessments of symptoms, signs, usage of medications and laboratory tests for renal and liver function [23]. Similarly, a stepwise risk stratification process for heart team decision-making for the treatment of TR has advocated assessing demographics, symptoms and comorbidities before cardiac pathological remodeling [24]. Here, we propose the baseline systemic characteristics harness identical importance to predict the prognosis of severe or greater TR patients. We suppose that advanced delineation of whether functional abnormalities in other systems are secondary to TR could enable us to re-evaluate patients before and after intervention so as to serve as an indicator for the effectiveness of treatment.

Nonetheless, our study has limitations inherent to single-center retrospective studies, including a small sample size, selection bias and the absence of core-laboratory adjudicated echocardiographic data. Additionally, follow-up was only conducted via telephone, which may have led to bias regarding the composite. The abnormalities identified cannot be perfectly considered as secondary to TR although we have made efforts to exclude patients with a clear diagnosis of comorbidities that could explain the corresponding abnormalities. We also must acknowledge that an increasing burden of comorbidities conveys a higher risk even without being causally related to TR. Moreover, the etiology of TR was not specified, which adds confounding factors to the classification and outcome analysis. Due to the restrictions of our electronic medical records system, we did not retrieve detailed echocardiographic measurements of this patient cohort, thus information such as left and right ventricular structures and functions could not be obtained in the present study. The criteria used in each system were limited by the availability of examinations during hospitalization and materials existed in our electronic medical records. The findings are subject to the time period of study and the setting of a tertiary center, thus the generality of the findings needs to be testified.

## Conclusions

Approximately 50% of patients with at least severe TR presented with two or more extra-cardiac manifestations in the scope of respiratory, hepatic and digestive, renal, hematopoietic and dermatic systems. The involvement of at least two extra-cardiac systems may incur an approximate of 2-fold higher risk (at borderline statistical significance) of all-cause death or cardiac rehospitalization than TR patients with one or less extra-cardiac involvement. A routine assessment of extra-cardiac manifestations from TR could add value to patient referral and the selection for definitive TR interventions.

**Table 1:** Baseline characteristics and prognosis of the cohort

	Overall (*n* = 258)	Stage 1 (*n* = 53)	Stage 2 (*n* = 71)	Stage 3 (*n* = 97)	Stage 4 (*n* = 37)	P value
Age, years	73 (62–83)	72 (59–83)	70 (63–82)	74 (63–84)	69 (54–79)	0.564
Female, n (%)	135 (52.3)	29 (21.5)	45 (33.3)	46 (34.1)	15 (11.1)	0.086
Severe TR, n (%)	239 (92.6)	53 (22.2)	66 (27.6)	84 (35.1)	36 (15.1)	0.114
More-than-severe TR, n (%)	19 (7.4)	0 (0)	5 (26.3)	13 (68.4)	1 (5.3)	
Atrial fibrillation, n (%)	158 (61.2)	31 (19.6)	44 (27.8)	74 (46.8)	9 (5.7)	**< 0.001**
Concomitant less than severe mitral valve disease, n (%)	162 (62.8)	33 (20.4)	44 (27.2)	69 (42.6)	16 (9.9)	**0.030**
Concomitant less than severe aortic valve disease, n (%)	75 (29.1)	14 (18.7)	16 (21.3)	39 (52.0)	6 (8.0)	**0.015**
Coronary artery disease, n (%)	40 (15.5)	5 (12.5)	15 (37.5)	12 (30.0)	3 (7.5)	0.181
Hypertension, n (%)	109 (42.4)	23 (21.1)	30 (27.5)	46 (42.2)	10 (9.2)	0.203
NT-proBNP, ng/L	1677 (930–3309)	1067 (513–2304)	1283 (597–2055)	2036 (1249–4756)	4072 (1865–8685)	**< 0.001**
Permanent pacemaker, n (%)	47 (18.2)	4 (8.5)	14 (29.8)	24 (51.1)	5 (10.6)	0.057
Prior stroke, n (%)	38 (14.7)	7 (18.4)	10 (26.3)	18 (47.4)	3 (7.9)	0.467
Typical heart failure symptoms and signs, n (%)	156 (60.5)	19 (12.2)	42 (26.9)	70 (44.9)	25 (16.0)	**< 0.001**
Ascites, n (%)	20 (7.8)	3 (15.0)	5 (25.0)	11 (55.0)	1 (5.0)	0.402
Chronic liver disease, n (%)	32 (12.4)	7 (21.9)	11 (34.4)	6 (18.8)	8 (32.0)	0.072
Chronic lung disease, n (%)	168 (65.1)	40 (23.8)	52 (31.0)	58 (34.5)	18 (10.7)	**0.017**
Chronic kidney disease, n (%)	77 (29.8)	5 (6.5)	14 (18.2)	48 (62.3)	10 (13.0)	**< 0.001**
Diabetes mellitus, n (%)	37 (14.3)	7 (18.9)	13 (35.1)	16 (43.2)	1 (2.7)	0.144
Use of diuretics, n (%)	188 (72.8)	34 (18.1)	57 (30.3)	81 (43.1)	16 (8.5)	**< 0.001**
Peripheral vascular disease, n (%)	47 (18.1)	8 (17.0)	10 (21.3)	25 (53.2)	4 (8.5)	0.100
^*^Composite endpoint in patients with severe TR, n (%)	40 (20.6)	6 (15.0)	12 (30.0)	15 (37.5)	7 (17.5)	0.256
^*^Composite endpoint in patients with more-than-severe TR, n (%)	7 (36.8)	0 (0)	1 (14.3)	5 (71.4)	1 (14.3)	

Categorical variables were shown as numbers (percentages) and continuous variables were shown as medians (25–75 percentiles)

TR: Tricuspid regurgitation; NT-proBNP: N-terminal-proB-type natriuretic peptide

**Bold values** are statistically significant

*Based on follow-up of 194 patients with severe TR and 19 patients with more-than-severe TR

## Data Availability

The datasets used and/or analyzed during the current study are available from the corresponding author on reasonable request.

## Abbreviations

TR: Tricuspid regurgitation
IQR: Interquartile range
OR: Odds ratio
CI: Confidence interval
hs-TnT: high-sensitivity troponin T
NT-proBNP: N-terminal-proB-type natriuretic peptide
ALT: Alanine aminotransferase
AST: Aspartate transaminase
eGFR: estimated glomerular filtration rate
